# Improved survival after early detection of asymptomatic distant metastasis in patients with thyroid cancer

**DOI:** 10.1038/s41598-019-55370-w

**Published:** 2019-12-10

**Authors:** Hosu Kim, So Young Park, Jaehoon Jung, Jung-Han Kim, Soo Yeon Hahn, Jung Hee Shin, Young Lyun Oh, Man Ki Chung, Hye In Kim, Sun Wook Kim, Jae Hoon Chung, Tae Hyuk Kim

**Affiliations:** 1 Division of Endocrinology & Metabolism, Department of Medicine, Thyroid Center, Samsung Medical Center, Sungkyunkwan University School of Medicine, Seoul, Korea; 2 Division of Endocrinology, Department of Medicine, Gyeongsang National University Changwon Hospital, Gyeongsang National University College of Medicine, Changwon, Korea; 3 Division of Breast and Endocrine Surgery, Department of Surgery, Samsung Medical Center, Sungkyunkwan University School of Medicine, Seoul, Korea; 4 Department of Radiology, Samsung Medical Center, Sungkyunkwan University School of Medicine, Seoul, Korea; 5 Department of Pathology and Translational Genomics, Samsung Medical Center, Sungkyunkwan University School of Medicine, Seoul, Korea; 6 Department of Otorhinolaryngology-Head and Neck Surgery, Samsung Medical Center, Sungkyunkwan University School of Medicine, Seoul, Korea; 7 Division of Endocrinology & Metabolism, Department of Medicine, Samsung Changwon Hospital, Sungkyunkwan University School of Medicine, Changwon, Korea

**Keywords:** Cancer screening, Thyroid diseases, Cancer screening

## Abstract

The incidence of thyroid cancer (TC) has been increasing in many countries and concerns about overdiagnosis are also widely shared. However, early detection may be helpful in some high-risk TC patients, such as those with initial distant metastasis. We conducted this study to evaluate the usefulness of early detection in TC patients with initial distant metastasis. We retrospectively reviewed the clinical data of 13,249 TC patients, and found 127 patients with initial distant metastasis. Enrolled patients were divided into two groups according to the diagnostic periods; before and after 2004, when the early detection of TC by ultrasonography began in earnest in Korea. Patients were also divided into two groups according to the presence of symptoms. Prior to 2004, 33 patients (1.7% of TC patients) were diagnosed with TC with initial distant metastasis and 16 (48.5%) of them died. After 2004, 94 patients (0.8% of TC patients) were diagnosed with TC with initial distant metastasis and 29 (30.9%) of them died. Prior to 2004, the disease-specific death rates were similar between the asymptomatic and symptomatic groups (46.2% vs. 50.0%, *P* = 0.566). Conversely, after 2004, the asymptomatic group showed a significantly lower disease-specific death rate as compared with that of the symptomatic groups (17.2% vs. 60.0%; *P* < 0.001). Early detection had a significant positive impact on survival outcomes only after 2004, especially in asymptomatic TC patients with initial distant metastasis.

## Introduction

In recent years, the incidence of thyroid cancer (TC) has been steadily increasing in many countries^1–3^. This trend is believed to be predominantly due to the rise in the prevalence of early detection of papillary thyroid carcinoma (PTC) less than 1 cm in diameter with high-resolution ultrasonography (US)^3–6^. However, because its age-adjusted survival rate was more than 100%, concern of overdiagnosis has been growing^3,7,8^.

Most patients with TC have a favorable prognosis^9,10^. However, the survival rate is significantly decreased when distant metastasis is detected at the time of diagnosis. Although distant metastases occur in small number of patients with TC, but represent the most frequent cause of thyroid cancer-related death^11^. Globally in 2012, estimated numbers of deaths from TC were 27,000 in women and 13,000 in men^12^. Long-term survival rates of differentiated TC (DTC) patients with initial distant metastasis range from 13% to 100% in previous studies^13–16^. The authors also reported that five-year and 10-year survival rates of DTC patients with initial distant metastasis are 85% and 68%, respectively^17,18^. Furthermore, TC patients with aggressive histology such as medullary thyroid carcinoma and anaplastic thyroid carcinoma have a poor prognosis^19–21^. Therefore, early detection and proper management are very important to improve the prognosis in TC patients with initial distant metastasis or aggressive histology^22^.

Recently, the authors reported the characteristics and prognoses of DTC patients with initial distant metastasis in Korea^17^. Survival rates in this study were higher than those in earlier studies (85% vs. 35% to 68%)^23,24^. In addition to PTC predominance and tyrosine kinase availability, involvement of small PTC less than 1 cm in this study might be a major cause of the observed dissimilarity. In other words, we assumed that early detection would be helpful in DTC patients with initial distant metastasis and would be associated with better prognosis. Therefore, we conducted the present study to evaluate the usefulness of early detection by US screening in TC patients with initial distant metastasis.

## Results

### Baseline characteristics

We evaluated 127 TC patients with initial distant metastasis. The proportion of initial distant metastasis was 1.0% (127/13,249) during the study period from 1994 to 2013. Of the 127 patients, 38 were diagnosed as distant metastasis through histological confirmation. Thirty-four patients underwent biopsy and four underwent surgery. Other patients were diagnosed as distant metastasis through radiological examination. The median age of these 127 individuals was 51 years (range: 13–77 years), and 69 patients (54.3%) were female. The participants were followed for a median of six years (range: 0–23 years). Among them, 119 patients (93.7%) and five patients (3.9%) had undergone total thyroidectomy and lobectomy, respectively. Three patients did not perform surgery due to advanced disease. Additionally, 32 patients (25.2%) and 61 patients (48.0%) had undergone only central neck dissection and central and lateral neck dissection, respectively. PTC was diagnosed in 67 patients (52.8%), FTC in 39 (30.7%), PDTC in seven (5.5%), MTC in eight (6.3%), and ATC in six (4.7%). Among 67 PTC patients, 61 were favorable histopathologic variants (53 were classic and eight were follicular variants PTC). Among the remaining six PTC patients, there were three diffuse sclerosing, one tall cell, one insular, and one oxyphilic variants. There were no statistically significant differences according to the histopathologic variants of PTC between before and after 2004 (*P* = 1.000 by chi-squared test). The median primary tumor size was 3.2 cm (interquartile range: 2.0–4.8 cm). Extrathyroidal extension and positive resection margin were found in 86 (67.7%) and 32 (25.2%) patients, respectively. Lymphatic and vascular invasion was detected in 35 (27.6%) and 21 (16.5%) patients, respectively. Of 106 patients with DTC, 100 received radioactive iodine (RAI) therapy. Twenty-one of them were refractory to RAI.

Forty-five (35.4%) patients died of thyroid carcinoma. Six out of 45 patients died of systemic diseases that worsened due to multiple bone metastases. Eight patients died of cord compression by spine metastasis, six due to complications caused by brain metastasis, 13 due to respiratory failure by lung metastasis, three due to hemoptysis by lung metastasis, five due to airway obstruction by tracheal invasion, three due to sepsis, and one from hepatic failure by liver metastasis. The five-year and 10-year cancer-specific survival rates were 70.9% and 55.6%.

### Differences in clinical characteristics according to the diagnostic periods

Before and after 2004, 33 and 94 patients were diagnosed with TC with initial distant metastasis, respectively, with diagnosis proportions being 1.7% (33/1,958) and 0.8% (94/11,291). There was a statistically significant difference in the route of detection between before and after 2004 (*P* = 0.007; Table 1). Prior to 2004, 13 affected patients (39.4%) belonged to the asymptomatic group, while 20 affected patients (60.6%) were identified by local or systemic symptoms. However, after 2004, 64 affected patients (68.1%) belonged to the asymptomatic group and 30 affected patients (31.9%) were identified by local or systemic symptoms. No clinical factors showed significant differences between before and after 2004, except neck LN dissection; specifically, after 2004, more neck LN dissection has been performed (*P* = 0.001; Table 1). In patients with DTC, RAI avidity was not statistically different between before and after 2004 (*P* = 0.09; Table 1). Furthermore, even if separating asymptomatic and symptomatic group, there were no significant statistical differences in clinical characteristics and disease status before and after 2004 (Supplementary Tables S1, S2).

**Table 1:** Characteristics of TC patients with initial distant metastasis before and after 2004.

Characteristics	Before 2004	After 2004	*P* value
Age at diagnosis (years)	44.2 ± 17.6	50.7 ± 18.4	0.075
Sex (male)	12 (36.4%)	46 (48.9%)	0.230
Route of detection			**0.007**
Asymptomatic screening	13 (39.4%)	64 (68.1%)	
Clinical local symptom	16 (48.5%)	20 (21.3%)	
Clinical systemic symptom	4 (12.1%)	10 (10.6%)	
Type of thyroid surgery			1.000
Total thyroidectomy	31 (96.9%)	89 (95.7%)	
Lobectomy	1 (3.0%)	4 (4.3%)	
LN dissection			**0.001**
No	16 (50.0%)	16 (17.2%)	
CND	3 (9.4%)	29 (31.2%)	
CND and LND	13 (40.6%)	48 (51.6%)	
Site of distant metastasis			0.434
Lung only	20 (60.6%)	47 (50.5%)	
Bone only	8 (24.2%)	22 (23.7%)	
Combined	5 (15.2%)	24 (25.8%)	
Tumor histology			0.672
PTC	18 (54.5%)	49 (52.1%)	
FTC	8 (24.2%)	31 (33.0%)	
PDTC	3 (9.1%)	4 (4.3%)	
MTC	3 (9.1%)	5 (5.3%)	
ATC	1 (3.0%)	5 (5.3%)	
Tumor size (cm)	3.5 ± 1.8	3.7 ± 2.6	0.605
Initial LN metastases >5	12 (36.4%)	43 (46.2%)	0.415
Positive lymphatic invasion	8 (25.0%)	27 (29.7%)	0.657
Positive blood vessel invasion	5 (15.6%)	16 (17.6%)	1.000
Positive resection margin	12 (38.7%)	20 (22.0%)	0.097
Positive ETE	25 (75.8%)	61 (65.6%)	0.384
RAI refractoriness^a^	2 (7.7%)	19 (25.7%)	0.090
T stage			0.721
T1	5 (15.2%)	23 (24.7%)	
T2	12 (36.4%)	31 (33.3%)	
T3	13 (39.4%)	31 (33.3%)	
T4	3 (9.1%)	8 (8.6%)	
N stage			0.393
N0	16 (48.5%)	33 (35.5%)	
N1a	4 (12.1%)	17 (18.3%)	
N1b	13 (39.4%)	43 (46.2%)	

Continuous data were given as medians ± standard deviations and categorical data were given as absolute numbers (percentages). Abbreviations: CND, central neck dissection; LND, lateral neck dissection; PTC, papillary thyroid carcinoma; FTC, follicular thyroid carcinoma; PDTC, poorly differentiated thyroid carcinoma; MTC, medullary thyroid carcinoma; ATC, anaplastic thyroid carcinoma; LN, lymph node; ETE, extrathyroidal extension.; RAI, radioactive iodine.

^a^Only for the DTC patients.

### Disease-specific death in thyroid cancer patients with initial distant metastasis

Of the total 127 patients, 45 patients (35.4%) died of TC during the follow-up period of a median of six years. Before and after 2004, 16 (48.5%) and 29 (30.9%) patients died of TC, respectively. Median follow-up periods were 13 and 5 years, five-year cancer-specific survival rates were 66.7% and 71.9%, respectively. Prior to 2004, six patients (46.2%) in the asymptomatic group and 10 (50.0%) in the symptomatic group died, respectively. However, after 2004, 11 patients (17.2%) in the asymptomatic group and 18 (60.0%) in the symptomatic group died.

### Prognosis of patients according to the time of diagnosis and route of detection

**Figure 1:**
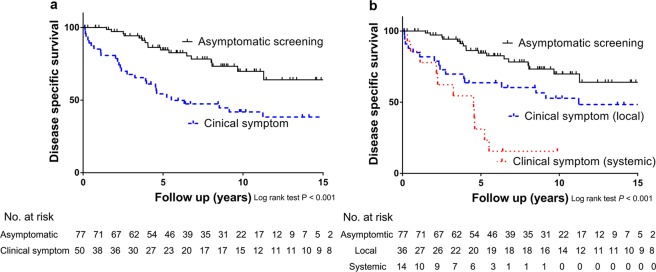
Analysis of disease specific death according to diagnostic method and diagnostic periods. (**a**) In the entire period, a diagnosis of TC and initial distant metastasis by asymptomatic screening was better than that with symptomatic diagnosis (*P* < 0.001). (**b**) When the diagnostic symptoms were divided into local and systemic symptoms, systemic symptoms had a poorer prognosis versus local symptoms (*P* < 0.001).

We performed the survival analysis according to the time of diagnosis (i.e., before 2004 and after 2004) and the route of detection. Over the entire period, the prognosis of the symptomatic group was worse than that of the asymptomatic group [HR: 2.98 (95% CI: 1.63–5.45); *P* < 0.001; Fig. 1a]. Among the patients of the symptomatic group, the prognosis of the systemic symptomatic group was worse than that of the local symptomatic group [HR: 2.23 (95% CI: 1.13–4.39) for local symptomatic group and HR: 5.94 (95% CI: 2.75–12.84) for systemic symptomatic group; *P* < 0.001; Fig. 1b].

**Figure 2:**
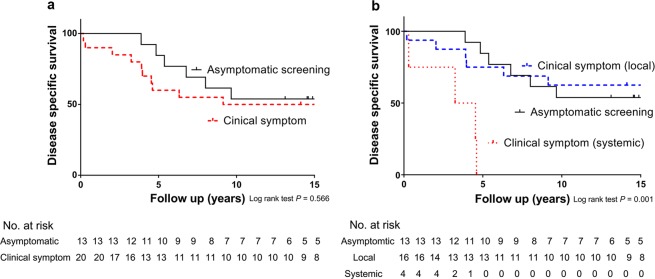
(**a**) Before 2004, the disease specific death of patients with TC and initial distant metastasis who were diagnosed by asymptomatic screening and clinical symptoms did not show a statistically significant difference (*P* = 0.566). (**b**) When the diagnostic symptoms were divided into local and systemic symptoms, only diagnosis with clinical symptoms showed a significantly poorer prognosis (*P* = 0.001).

**Figure 3:**
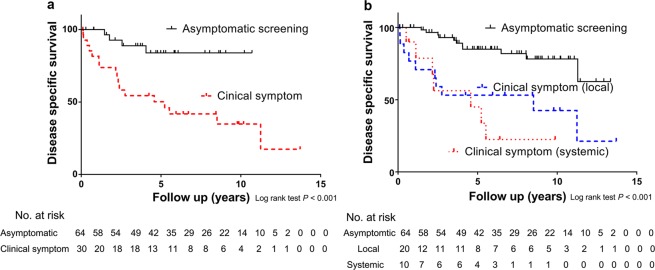
(**a**) After 2004, the disease specific death of patients with TC and initial distant metastasis who were diagnosed by asymptomatic screening and clinical symptoms showed a statistically significant difference (*P* < 0.001). (**b**) Asymptomatic screening showed a better prognosis than did systemic systems as well as local symptoms (*P* < 0.001).

Prior to 2004, the disease-specific survival rate of the asymptomatic group was not significantly different from that of the symptomatic group (*P* = 0.566; Fig. 2a). In more detail, the survival rate of the asymptomatic group was similar to that of the local symptomatic group, but was significantly better than that of the systemic symptomatic group [HR: 0.88 (95% CI: 0.28–2.73) for the local symptomatic group, *P* = 0.822; HR: 8.29 (95% CI: 1.95–35.19) for the systematic symptomatic group, *P* = 0.004; Fig. 2b]. However, after 2004, the disease-specific survival rate of the asymptomatic group was significantly better than that of the symptomatic group [HR: 4.94 (95% CI: 2.33–10.49); *P* < 0.001; Fig. 3a]. In more detail, the survival rate of the asymptomatic group was significantly better than those of the local and systemic symptomatic groups, respectively [HR: 4.38 (95% CI: 1.90–10.13) for the local symptomatic group, *P* = 0.001; HR: 6.22 (95% CI: 2.38–16.29) for the systematic symptomatic group, *P* < 0.001; Fig. 3b]. Following adjustment for other variables, the route of detection was significant after 2004 but not before 2004. In other words, after 2004, the risk for disease-specific death of the asymptomatic group was significantly lower than that of the symptomatic group [HR: 0.19 (95% CI: 0.06–0.60); *P* = 0.005; Table 2].

**Table 2:** Prognostic factors for disease specific death in TC patients with initial distant metastasis according to diagnosis period.

Characteristics	Before 2004		After 2004	
	Hazard ratio (95% CI)	*P* value	Hazard ratio (95% CI)	*P* value
Age at diagnosis (years)	1.05 (0.94–1.16)	0.379	1.07 (1.03–1.12)	**0.001**
Sex (male)	0.35 (0.01–13.21)	0.568	1.23 (0.27–5.61)	0.788
LN dissection				
No	Reference	0.998	Reference	0.526
CND	0.00 (0.00–0.00)	0.988	0.99 (0.20–4.92)	0.988
CND and LND	1.12 (0.02–70.69)	0.956	0.30 (0.02–4.92)	0.343
Poor tumor histology	9.03 (0.42–194.50)	0.160	17.31 (3.15–95.12)	**0.001**
Tumor size	1.63 (0.88–3.04)	0.122	0.85 (0.67–1.06)	0.147
Initial LN metastases > 5	0.07 (0.00–1.95)	0.117	4.02 (0.52–31.14)	0.183
Positive lymphatic invasion	0.87 (0.06–11.57)	0.913	0.57 (0.17–1.93)	0.366
Positive blood vessel invasion	1.69 (0.03–102.52)	0.803	0.84 (0.17–4.19)	0.833
Positive resection margin	0.52 (0.03–9.64)	0.659	1.21 (0.33–4.52)	0.772
Positive ETE	0.71 (0.08–6.68)	0.765	0.69 (0.21–2.27)	0.542
Site of distant metastasis				
Lung only	Reference	0.527	Reference	**0.033**
Bone only	0.56 (0.03–10.78)	0.698	0.43 (0.07–2.61)	0.357
Combined	6.76 (0.12–379.88)	0.353	2.91 (0.72–11.65)	0.132
Route of detection				
Asymptomatic screening	0.28 (0.02–3.85)	0.342	0.19 (0.06–0.60)	**0.005**

Cox proportional hazards regression model was performed. Abbreviations: CI, confidence interval; LN, lymph node; CND, central neck dissection; LND, lateral neck dissection; LN, lymph node; ETE, extrathyroidal extension. Poor tumor histology mean PDTC, MTC, and ATC.

### Subgroup analysis according to the histologic type of thyroid cancer

There were 106 DTC patients and 28 of them died of the disease. Asymptomatic screening and symptomatic group did not show a statistically significant difference in disease specific death before 2004. However, asymptomatic group showed a significantly improved prognosis only after 2004 (*P* = 0.981 for before 2004 and *P* = 0.012 for after 2004 years, respectively. By log-rank test.) For PDTC, MTC and ATC, survival analysis could not performed because of the small number of patients in each subgroup (only two patients were diagnosed by asymptomatic screening).

## Materials and Methods

### Patients

We reviewed the clinical data of 13,249 patients with TC who underwent thyroidectomy between 1994 and 2013 at Samsung Medical Center in Seoul, Korea. Among them, we identified 127 patients with initial distant metastasis at the time of diagnosis. All patients with PTC, follicular thyroid carcinoma (FTC), poorly-differentiated thyroid carcinoma (PDTC), medullary thyroid carcinoma (MTC), and anaplastic thyroid carcinoma (ATC) were included. Initial distant metastasis was defined as distant metastasis detected prior to or within six months of the initial thyroidectomy. Distant metastasis was found by pathological confirmation or imaging studies such as whole-body scan (WBS) or computed tomography (CT), magnetic resonance imaging (MRI), bone scan, or positron-emission tomography (PET) scans. Patients were treated with the American Thyroid Association guidelines consistent with the each era. This study was approved by the institutional review board (IRB) of Samsung Medical Center (IRB no. SMC2017-02-058), and performed in accordance with relevant guidelines and regulations. The IRB waived the requirement for informed consent because all patient data was de-identified.

### Study design

The enrolled 127 patients were divided into two groups according to the diagnostic periods of before and after 2004, respectively, as 2004 was when the early detection of TC by US began in earnest in Korea^3^. Patients were also divided into three groups according to the presence of symptoms, as follows: asymptomatic, local symptoms, and systemic symptoms. The route of detection for TC in the asymptomatic group was as follows: 1) by US screening in subjects for a health promotion program 2) by imaging studies for other diseases, including other malignancies or benign thyroid diseases; and 3) by analysis of surgical specimens from thyroid surgery for benign thyroid diseases. Patients classified into the local symptoms group were diagnosed by palpation of a neck mass or presentation of neck discomfort or hoarseness. Patients placed in the systemic symptoms group complained of dyspnea, hemoptysis, dysphagia, bone pain, and weight loss.

Data on age at the time of diagnosis as well as sex, histologic type, primary tumor size, extrathyroidal extension, lymph node (LN) metastasis, involvement of resection margin, and lymphovascular invasion were included for analysis of prognosis. Disease-specific death events and survival rate were evaluated to compare the clinical outcomes. The eighth edition of the tumor node metastasis staging system was used to define cancer stage.

### Statistical analysis

For statistical analysis, continuous data were expressed in the format of mean ± standard deviation. Data on categorical characteristics were expressed as percent values or absolute numbers. For comparisons of clinical and pathological characteristics between the patients groups, a chi-squared test was used for categorical data, while a *t*-test was used for continuous data. A Kaplan–Meyer curve and a log-rank test were used for survival analysis. A Cox proportional hazards model applying the input method was used to identify factors associated with disease-specific death. Hazard ratios (HRs) and 95% confidence intervals (CIs) were used. A *P* value of < 0.05 was considered to be significant. Statistical analysis was performed using the Statistical Package for the Social Sciences software version 23 (IBM Corp., Armonk, NY, USA).

## Discussion

Early detection was associated with a decreased proportion of TC patients with initial distant metastasis. Moreover, it had a significant positive impact on the survival outcomes, especially in asymptomatic TC patients with initial distant metastasis. The results of this study contradict the prevailing perception that TC screening is more harmful than beneficial. The data presented here suggest that less is not always better.

The incidence of TC has been increasing in many countries as well as in Korea. This is mainly due to the increase in early detection of PTC less than 1 cm in diameter with the introduction of high-resolution US. In Korea, the screening of TC by US began in earnest in 2004. Our data showed that the number of patients with TC has increased, but the proportion of patients with initial distant metastasis has decreased since 2004. This is because the number of patients with initial distant metastasis has increased, but the number of patients with early-stage disease has also grown more since 2004. Therefore, the explosive increase in early TC caused concern about overdiagnosis^3,4^. However, “cancer” consists of complex and various stages of disease^17,25^. Early detection may be helpful in patients with advanced TC, such as initial distant metastasis. Unfortunately, to our knowledge, there have been no studies related to it conducted to date.

In this study, the prognosis of asymptomatic TC patients with initial distant metastasis diagnosed since 2004 was much improved as compared with prior to 2004 (Figs. 2a and 3a). This suggests that the increased sensitivity and wide use of screening US has strengthened the ability to improve prognosis rather than symptomatic diagnosis. Early detection led to an improvement of the prognosis of the asymptomatic screening group as compared with the symptomatic group after 2004 (Fig. 3b). This result was statistically significant even following adjustment for other factors (Table 2).

There are several hypotheses about the nature of screening method used to improve the prognosis of TC patients with initial distant metastasis. First, there might be a stage migration effect^26^. Early advanced TC, such as initial distant metastasis, can be detected earlier by the developed screening method and thus potentially preemptively managed. In our previous study, distant metastasis was composed of various disease groups that had different prognoses^17^. For example, multiple-site distant metastasis had a poorer prognosis than did lung metastasis. Furthermore, among types of lung metastasis, macronodular metastasis had a worse prognosis versus micronodular metastasis. Although distant metastasis is a progressive disease, it is also a diverse group of diseases and some of the conditions can be treated if diagnosed early. Therefore, we postulated that stage migration occurred. Park *et al*. reported that the proportion of TC patients with distant metastasis was decreasing in Korea^3^. Our data also showed that the proportion of distant metastasis has decreased since 2004 in comparison with the period before 2004. A multicenter cohort study in Korea reported that the mortality rate of TC has decreased over time^8^. Therefore, it can be postulated that the survival rate of TC has been improving in Korea, because patients with advanced stages or distant metastases who were not identified in the past were diagnosed by a new diagnostic technique. Second, the early detection of advanced patients enabled us to treat these individuals more aggressively. More prevalent detection of TC has increased the frequency of thyroid surgery and has led to the production of competent, high-volume surgeons in the tertiary care centers. In our previous study, the prognosis of advanced TC performed by high-volume surgeons was better than that performed by low-volume surgeons^27^. Our data showed that, after 2004, neck dissection was significantly performed more than prior to 2004. In addition, after 2004, additional treatment such as systemic chemotherapy, radiotherapy, and tyrosine kinase inhibitor (TKI) administration was performed more than before 2004 (50% vs. 36%). When analyzed only for TKI treatment, more patients were treated with TKI after 2004 than before 2004 (17% vs. 6%). In our recent study, asymptomatic patients with distant metastasis were more likely to respond to sorafenib than symptomatic patients^28^. Therefore, we can postulate that the development of a screening method could identify an ideal time for TKI therapy and enable early aggressive therapy in TC patient with initial distant metastasis. Third, socioeconomic status may be related to the prognosis of the asymptomatic screening group. The age-standardized mortality rate of TC in Korea is more than 100%^7^. This suggested that people who were diagnosed and treated with TC had better health care than those who were not. A previous study reported that insurance coverage affected the prognosis of cancer patients^29,30^. Therefore, people who get screening for TC are more likely to take better care of their health.

The present study had several notable strengths. First, this study revealed that early detection by screening was helpful in some groups of TC. To date, the development of screening in TC has a concern of overdiagnosis. However, this study found that the developed screening method could improve the prognosis of high-risk TC patients such as those with initial distant metastasis. Second, this study included a relatively large number of patients with initial distant metastasis. Most TCs are diagnosed as localized disease and initial distant metastasis is not common. Third, this analysis was divided into the two periods of before and after 2004, which was when the early detection of TC by US began in earnest in Korea. Therefore, we were able to elucidate the effects of screening on the prognosis of TC with initial distant metastasis in more detail.

This study also had several limitations. First, there may be a lead time bias associated with improved survival in screening group, because of an earlier point of diagnosis from which survival is measured^31^. This bias is inevitable in a retrospective design, which is a nonrandomized evaluation. To overcome this inherent limitation, long-term randomized trial should compare mortality from the TC in the entire screening group with that in the unscreened group. Second, although this study included a relatively high number of TC patients with initial distant metastasis, the absolute number of patients was too small to perform various subgroup analyses. Large-scale multicenter studies are needed to validate current results. Third, it is difficult to clarify causal relationship by retrospective study design. Fourth, this study was conducted in a single tertiary care center, and it might have selection bias.

In summary, although the development of a screening test has led to an explosive increase in the incidence of TC after 2004, early detection was shown to substantially reduce the risk of dying from TC in patients with initial distant metastasis. Since imprudent use of imaging tests in the general population is not associated with the survival benefit of TC^3^, further large population research should pursue how to select individuals who might have a timely benefit while minimizing the potential harms of screening.

## Supplementary information

Supplementary Table

## Data Availability

The datasets generated during and/or analyzed during the current study are available from the corresponding author on reasonable request.
