# Supplementary material for: Improved survival after early detection of asymptomatic distant metastasis in patients with thyroid cancer
Source: Sci Rep. 2019 Dec 10;9:18745. doi: 10.1038/s41598-019-55370-w (PMC6904730; doi:10.1038/s41598-019-55370-w)
Supplement: Supplementary file 1 — Supplementary Table [file 41598_2019_55370_MOESM1_ESM.docx]

**Improved survival after early detection of asymptomatic distant metastasis in patients with thyroid cancer**

**Abbreviated title**: Early detection of metastatic thyroid cancer

Hosu Kim^1,2^, So Young Park^1^, Jaehoon Jung^2^, Jung-Han Kim^3^, Soo Yeon Hahn^4^, Jung Hee Shin^4^, Young Lyun Oh^5^, Man Ki Chung^6^, Hye In Kim^7^, Sun Wook Kim^1^, Jae Hoon Chung^1^ & Tae Hyuk Kim^1^

^1^Division of Endocrinology & Metabolism, Department of Medicine, Thyroid Center, Samsung Medical Center, Sungkyunkwan University School of Medicine, Seoul, Korea;

^2^Division of Endocrinology, Department of Medicine, Gyeongsang National University Changwon Hospital, Gyeongsang National University College of Medicine, Changwon, Korea;

^3^Division of Breast and Endocrine Surgery, Department of Surgery, Samsung Medical Center, Sungkyunkwan University School of Medicine, Seoul, Korea;

^4^Department of Radiology, Samsung Medical Center, Sungkyunkwan University School of Medicine, Seoul, Korea;

^5^Department of Pathology and Translational Genomics, Samsung Medical Center, Sungkyunkwan University School of Medicine, Seoul, Korea;

^6^Department of Otorhinolaryngology-Head and Neck Surgery, Samsung Medical Center, Sungkyunkwan University School of Medicine, Seoul, Korea;

^7^Division of Endocrinology & Metabolism, Department of Medicine, Samsung Changwon Hospital, Sungkyunkwan University School of Medicine, Changwon, Korea.

**Corresponding authors**

Correspondence to:

Tae Hyuk Kim, MD, PhD

Division of Endocrinology & Metabolism, Department of Medicine, Thyroid Center, Samsung Medical Center, Sungkyunkwan University School of Medicine 81 Irwon-ro, Gangnam-gu, Seoul 06351, Korea

Tel: +82-2-3410-6049; Fax: +82-2-3410-3849

E-mail: taehyukmd.kim@samsung.com

Co-correspondence to:

Jae Hoon Chung, MD, PhD

Division of Endocrinology & Metabolism, Department of Medicine, Thyroid Center, Samsung Medical Center, Sungkyunkwan University School of Medicine

81 Irwon-ro, Gangnam-gu, Seoul 06351, Korea

Phone: +82-2-3410-3434; Fax: +82-2-3410-3849

E-mail: thyroid@skku.edu

**Supplementary Table S1. Difference of clinical characteristics of asymptomatic patients before and after 2004.**

| Characteristics | Before 2004 | After 2004 | *P* value |
| --- | --- | --- | --- |
| Age at diagnosis (years) | 42.6 ± 14.5 | 51.3 ± 18.9 | 0.122 |
| Sex (male) | 3 (23.1%) | 31 (48.4%) | 0.129 |
| Site of distant metastasis |  |  | 0.870 |
| Lung only | 8 (61.5%) | 36 (56.3%) |  |
| Bone only | 3 (23.1%) | 14 (21.9%) |  |
| Combined | 2 (15.4%) | 14 (21.9%) |  |
| Tumor histology |  |  | 0.110 |
| PTC | 8 (61.5%) | 39 (60.9%) |  |
| FTC | 3 (23.1%) | 23 (35.9%) |  |
| PDTC | 1 (7.7%) | 2 (3.1%) |  |
| MTC | 1 (7.7%) | 0 (0.0%) |  |
| Tumor size (cm) | 3.6 ± 2.0 | 3.3 ± 2.3 | 0.673 |
| Initial LN metastases > 5 | 4 (30.8%) | 30 (46.9%) | 0.366 |
| Positive lymphatic invasion | 2 (15.4%) | 16 (25.0%) | 0.721 |
| Positive blood vessel invasion | 1 (7.7%) | 8 (12.5%) | 1.000 |
| Positive resection margin | 12 (38.7%) | 20 (22.0%) | 0.097 |
| Positive ETE | 9 (69.2%) | 42 (65.6%) | 1.000 |
| T stage |  |  | 0.626 |
| T1 | 3 (23.1%) | 18 (28.1%) |  |
| T2 | 4 (30.8%) | 24 (37.5%) |  |
| T3 | 6 (46.2%) | 19 (29.7%) |  |
| T4 | 0 (0%) | 3 (4.7%) |  |
| N stage |  |  | 0.174 |
| N0 | 6 (46.2%) | 22 (34.4%) |  |
| N1a | 4 (30.8%) | 10 (15.6%) |  |
| N1b | 3 (23.1%) | 32 (50.0%) |  |

Continuous data were given as medians ± standard deviations and categorical data were given as absolute numbers (percentages). Abbreviations: PTC, papillary thyroid carcinoma; FTC, follicular thyroid carcinoma; PDTC, poorly differentiated thyroid carcinoma; MTC, medullary thyroid carcinoma; ATC, anaplastic thyroid carcinoma; LN, lymph node; ETE, extrathyroidal extension

**Supplementary Table S2. Difference of clinical characteristics of symptomatic patients before and after 2004.**

| Characteristics | Before 2004 | After 2004 | *P* value |
| --- | --- | --- | --- |
| Age at diagnosis (years) | 45.2 ± 19.7 | 49.4 ± 17.6 | 0.425 |
| Sex (male) | 9 (45.0%) | 15 (50.0%) | 0.779 |
| Site of distant metastasis |  |  | 0.434 |
| Lung only | 20 (60.6%) | 47 (50.5%) |  |
| Bone only | 8 (24.2%) | 22 (23.7%) |  |
| Combined | 5 (15.2%) | 24 (25.8%) |  |
| Tumor histology |  |  | 0.672 |
| PTC | 18 (54.5%) | 49 (52.1%) |  |
| FTC | 8 (24.2%) | 31 (33.0%) |  |
| PDTC | 3 (9.1%) | 4 (4.3%) |  |
| MTC | 3 (9.1%) | 5 (5.3%) |  |
| ATC | 1 (3.0%) | 5 (5.3%) |  |
| Tumor size (cm) | 3.5 ± 1.8 | 4.7 ± 2.9 | 0.108 |
| Initial LN metastases > 5 | 8 (40.0%) | 13 (44.8%) | 0.777 |
| Positive lymphatic invasion | 6 (31.6%) | 11 (40.7%) | 0.555 |
| Positive blood vessel invasion | 4 (21.1%) | 8 (29.6%) | 0.735 |
| Positive resection margin | 12 (38.7%) | 20 (22.0%) | 0.097 |
| Positive ETE | 16 (80.0%) | 19 (65.5%) | 0.344 |
| T stage |  |  | 0.667 |
| T1 | 2 (10.0%) | 5 (17.2%) |  |
| T2 | 8 (40.0%) | 7 (24.1%) |  |
| T3 | 7 (35.0%) | 12 (41.4%) |  |
| T4 | 3 (15.0%) | 5 (17.2%) |  |
| N stage |  |  | 0.060 |
| N0 | 10 (50.0%) | 11 (37.9%) |  |
| N1a | 0 (0%) | 7 (24.1%) |  |
| N1b | 10 (50.0%) | 11 (37.9%) |  |

Continuous data were given as medians ± standard deviations and categorical data were given as absolute numbers (percentages). Abbreviations: PTC, papillary thyroid carcinoma; FTC, follicular thyroid carcinoma; PDTC, poorly differentiated thyroid carcinoma; MTC, medullary thyroid carcinoma; ATC, anaplastic thyroid carcinoma; LN, lymph node; ETE, extrathyroidal extension
